# Sources, Transformations, Syntheses, and Bioactivities of Monoterpene Pyridine Alkaloids and Cyclopenta[c]pyridine Derivatives

**DOI:** 10.3390/molecules27217187

**Published:** 2022-10-24

**Authors:** Xuejian Zhang, Feiyan Tao, Tao Cui, Cheng Luo, Zhigang Zhou, Yuchuan Huang, Lanlan Tan, Wei Peng, Chunjie Wu

**Affiliations:** 1 Research and Development Centre, China Tobacco Sichuan Industrial Co., Ltd., Chengdu 610066, China; 2 Sichuan Sanlian New Material Co., Ltd., Chengdu 610041, China; 3 Harmful Components and Tar Reduction in Cigarette Sichuan Key Laboratory, Chengdu 610066, China; 4 School of Pharmacy, Chengdu University of Traditional Chinese Medicine, Chengdu 611137, China

**Keywords:** monoterpene pyridine alkaloids, cyclopenta[c]pyridine derivatives, source, transformation, synthesis, bioactivity

## Abstract

Monoterpene pyridine alkaloids (MTPAs) are alkaloids derived from iridoid glycosides (IGs). The common molecular structure of MTPAs is the pyridine ring, while some of them have a cyclopenta[c]pyridine skeleton. Some compounds containing this structure are potentially bioactive medicinal agents. In this paper, seven drug candidates (**A**–**G**), ninety natural source products (**1**–**90**), thirty-seven synthesized compounds (**91**–**127**), as well as twenty-six key intermediates (**S1**–**S26**) were summarized. We categorized five types of MTPAs and one type of cyclopenta[c]pyridine alkaloids in all. Additionally, their possible genetic pathways were proposed. Then, the chemical transformation, biotransformation, chemical synthesis, as well as the bioactivity of MTPAs and cyclopenta[c]pyridine derivatives were analyzed and summarized. Cyclopenta[c]pyridine derivatives can be concisely and chirally synthesized, and they have shown potentials with antibacterial, insecticidal, antiviral, anti-inflammatory, and neuropharmacological activities.

## 1. Introduction

**Scheme 1:**
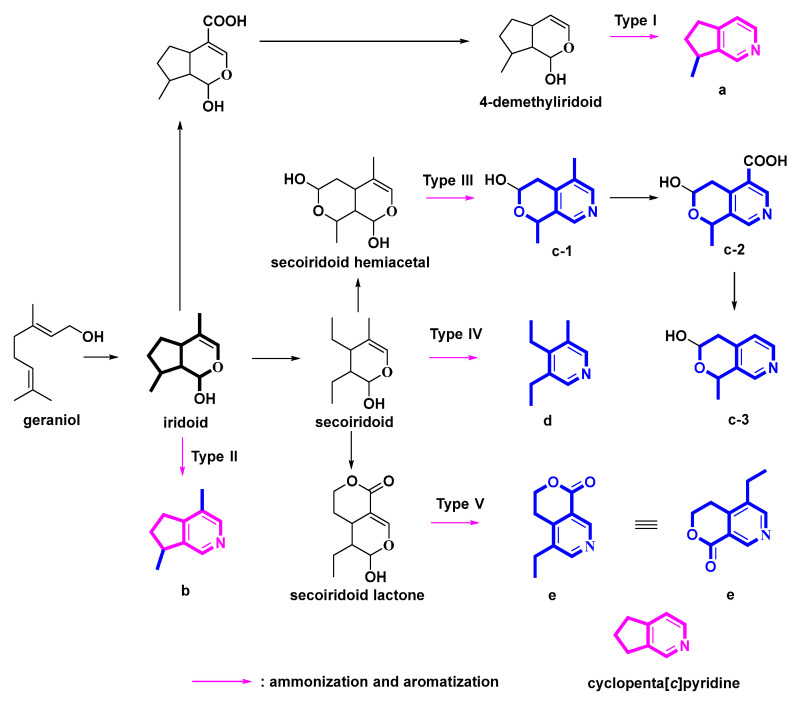
Possible genetic pathways from iridoids to MTPAs.

**Scheme 2:**
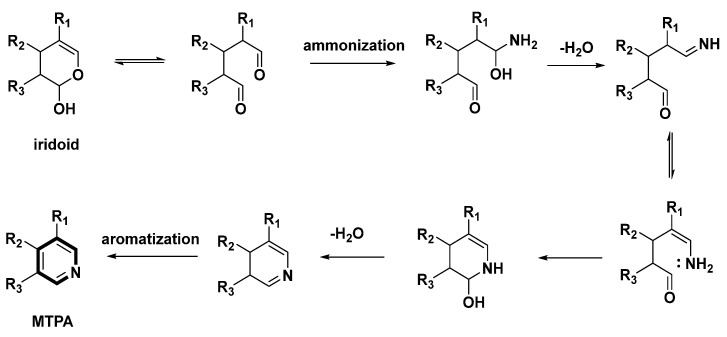
Possible ammonization and aromatization pathway from iridoids to MTPAs.

The nitrogen atom in pyridines, which are prized scaffolds in medicinal chemistry, is critical to the pharmacological profile of many medications that contain this heterocycle [1]. All monoterpene pyridine alkaloids (MTPAs) have a pyridine structure, most of which possessed a cyclopenta[c]pyridine molecular skeleton. Monoterpenes, mostly iridoid glycosides (IGs), are presumed to be biological or chemical synthetic precursors of MTPAs [2]. IGs are a class of substances with a structure resembling iridodial (Scheme 1 and Scheme 2), a chemical frequently used by plants as a defensive component.

**Figure 1:**
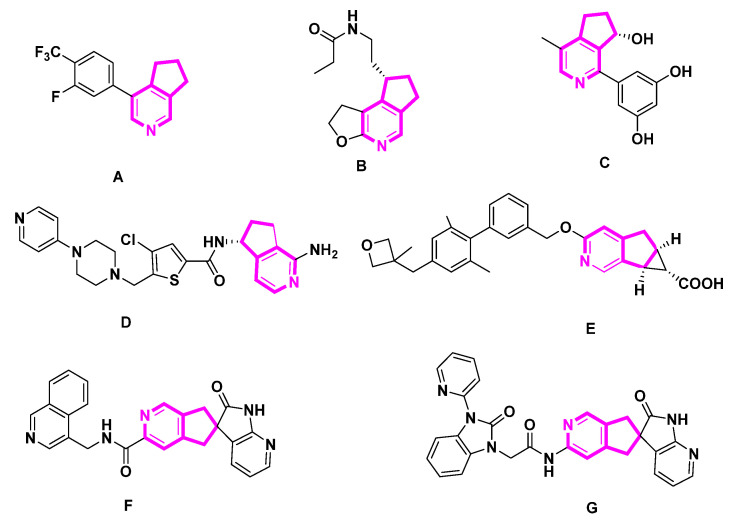
Examples of bioactive cyclopenta[c]pyridine derivatives.

Alkaloids are nitrogenous heterocyclic metabolites characterized by their structural diversity and bioactivity. Alkaloids are fundamental in organic chemistry and synthetic drug discovery. The pyridine structure in MTPAs is a functional molecular backbone widely found in natural products and bioactive molecules. The cyclopenta[c]pyridine or pyridine structure is a key fragment in a large proportion of bioactive compounds. Compounds containing cycloalkane pyridine have been used as intermediates in the synthesis of alkaloids or precursors of bioactive agents [3] (Figure 1). Compound **A** is a highly selective and potent aldosterone synthase inhibitor with an IC_50_ of 1 nM [4]. Ramelteon B’s 4-aza counterpart is a strong melatonin receptor agonist [5]. Additionally, the naturally occurring alkaloid sinensine C has shown cytoprotective action that may be helpful [5]. Compound **D** exhibited FXIIa inhibitory activity with an IC_50_ value in the range from 10 µM to 40 µM [6]. As a G-protein coupled receptor 40 (GPR40) agonist, compound **E** may be helpful in the treatment, suppression, and prevention of illnesses mediated by GPR40, including type 2 diabetes mellitus [7]. With IC_50_ values under 50 µM, compounds **F** and **G** are calcitonin gene-related peptide (CGRP) receptor antagonists that may be helpful in the treatment or prevention of CGRP-associated illnesses such as migraine or cluster headaches [8,9]. As a result, cyclopenta[c]pyridine-containing bioactive compounds are important research targets.

Due to the significance of the chemistry and bioactivity of MTPAs and cyclopenta[c]pyridine alkaloids, we focus on the structures of MTPAs and cyclopenta[c]pyridines, as well as their sources, chemical synthesis, and bioactivities in this review.

## 2. MTPAs and Their Activities

According to the different molecular skeletons and genetic pathways, we classified five types of transformation pathways from IGs to MTPAs. Five types were presented in Scheme 1: pyridine alkaloids derived from 4-demethyliridoids (Type I), pyridine alkaloids derived from iridoids (Type II), pyridine alkaloids derived from hemiacetal secoiridoids (Type III), pyridine alkaloids derived from secoiridoids (Type IV), and pyridines alkaloids derived from lactone secoiridoids (Type V). In addition, the phenyl-substituted cyclopenta[c]pyridine skeleton specifically existed in the genus Ganoderma (Type VI). In sum, Type I-V pyridine derivatives all originated from monoterpenoids. The Type I, II, and VI derivatives possessed the common cyclopenta[c]pyridine skeleton. Accordingly, this review has been organized in this manner. The category details in the following sections could help us obtain a clear picture of MTPAs and cyclopenta[c]pyridines, along with their origins, structures, sources, and bioactivities.

The ammonization and aromatization pathways from iridoids to pyridines were arranged and proposed (Scheme 2). Geraniol is an important precursor of MTPAs. Then, hydroxylation, oxidation and cyclization reactions on geraniol yield the intermediate iridoids [10]. Oxidation and hemiacetal formation lead to the production of the heterocyclic ring of iridoids [10]. Further ammonization and oxidation could provide the pyridine ring as follows (Scheme 2). At the beginning, secoiridoids are ammonified and dehydrated to afford enamines. Subsequently, nucleophilic addition/aromatization reactions of enamines could yield the pyridine ring.

### 2.1. Pyridine Alkaloids Derived from 4-Demethyliridoids (Figure 2, Table 1, Type I)

4-Demethypyridine alkaloids, processing the 8-methylcyclopenta[c]pyridine skeleton, are derived from iridoid through the oxidation of 4-methyl, decarboxylation of 4-carboxyl, ammonization, and aromatization (Scheme 1, Type I). In this section, nineteen 4-demethypyridine derivatives from plants are described, including three dimers (**17**–**19**).

Scrophularianine A (**1**), B (**2**), and C (**3**) were extracted and isolated from *Scrophularia ningpoensis* without using acids, bases, or nitrogen-containing salts [10]. In this case, these monoterpene alkaloids were thought to be natural MTPAs. MTPAs are mainly structurally related to iridoid compounds with the oxygen heterocycle being replaced by the pyridine ring.

**Figure 2:**
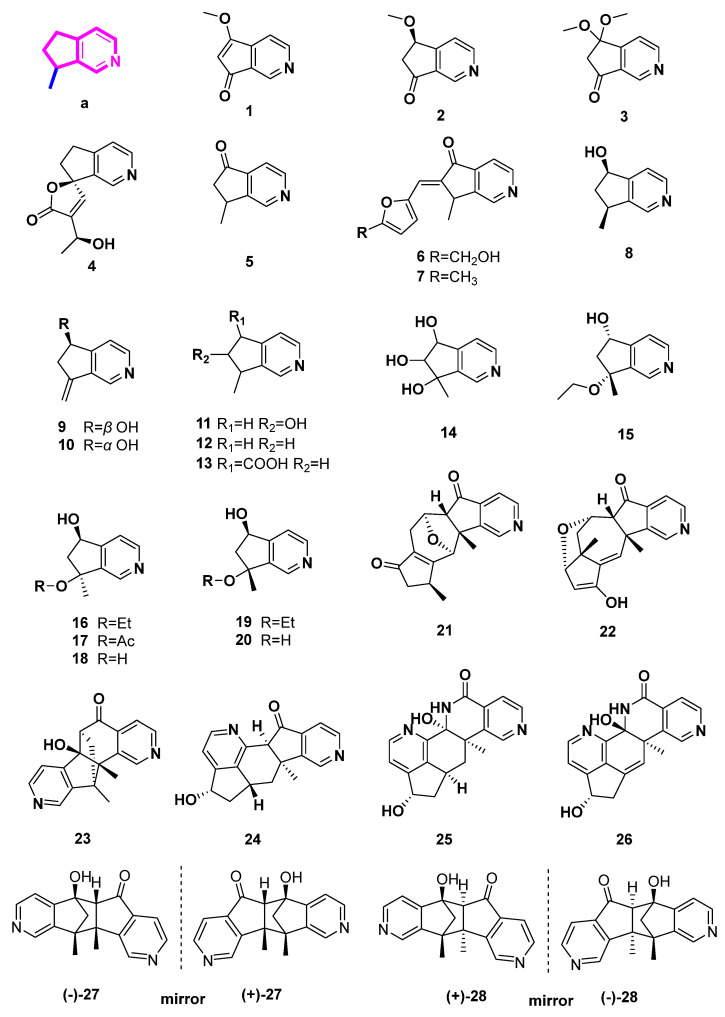
Structures of pyridine alkaloids derived from 4-demethyliridoids (Type I).

Plumerianine (**4**) was isolated from *Plumeria acutifolia* (Apocynaceae), using aqueous ammonia for alkaloid extraction. The iridoid glycosides from *P. acutifolia* were unsaturated at C-6 and C-7, while plumerianine was saturated at C-6 and C-7. Therefore, the author believed that plumerianine (**4**) was not an artifact from iridoid glycosides, but rather a natural product [11].

The primary iridoids of *Harpagophytum procumbens* or *Harpagophytum zeyheri*, as well as harpagide, harpagoside, and 8-O-p-coumaroylharpagide were treated with NH_3_ and HCl to produce aucubinine B (**5**). A brown residue was produced by commercially available *H. procumbens* extract that had been first treated with ammonia and subsequently with hydrochloric acid. Aucubinine B (**5**), beatrine A (**6**), and beatrine B (**7**) were produced and separated from this residue [12]. Aucubinine B (**5**), coelobillardierine (**8**), and 7,8-dehydro-coelobillardierine (**9**) were obtained from ammonified extracts of *Ceolospermun billardieri* with NH_4_OH basification [13]. In a similar way, cantleyine I (**11**), 4-noractinidine (**12**), and pedicularine (**13**) were incompletely characterized from *Castilleja miniate* (Scrophulariaceae) and *Penstemon whippleanus* (Scrophulariaceae), both of which were the host plants for the plume moth *Amblyptilia (Platyptifia) pica* (Walsingham) [14]. In another case, Salviadiginine A (**14**) was isolated from the roots of *Salvia digitaloids* [15].

**Table 1:** Pyridine alkaloids derived from 4-demethyliridoids (Type I).

Number	Compound Name	Source	Reference
** 1 **	scrophularianine A	* Scrophularia ningpoensis *	[10]
** 2 **	scrophularianine B	* Scrophularia ningpoensis *	[10]
** 3 **	scrophularianine C	* Scrophularia ningpoensis *	[10]
** 4 **	plumerianine	* Plumeria acutifolia *	[11]
**5**	aucubinine B	*Harpagophytum procumbens*, *Ceolospermum billardieri*, *Caryopteris mongolica* Bunge with basification of NH_4_OH	[12,13]
**6**	beatrine A	*Harpagophytum procumbens*	[12]
** 7 **	beatrine B	* Harpagophytum procumbens *	[12]
** 8 **	coelobillardine	* Ceolospermum billardieri *	[13]
** 9 **	dehydro 7-8 coelobillardierine	* Ceolospermum billardieri *	[13]
** 10 **	(*S*)-7-Methylene-6,7-dihydro-5*H*-cyclopenta[c]pyridine-5-ol	* Caryopteris glutinosa * with basification of NH_4_OH	[16]
** 11 **	cantleyine I	* Castilleja miniata *	[14]
** 12 **	4-noractinidine	* Penstemon whippleanus *	[14]
** 13 **	pedicularine	* Penstemon whippleanus *	[14]
** 14 **	salviadiginine A	* Salvia digitaloids *	[15]
** 15 **	(5S*,7R*)-7-ethoxy-6,7-dihydro-7-methyl-5H-cyclopenta[c]pyridin-5-ol	* Caryopteris mongolica * Bunge with basification of NH_4_OH	[17]
** 16 **	caryopterisines F	* Caryopteris glutinosa * with basification of NH_4_OH	[16]
** 17 **	caryopterisines G	* Caryopteris glutinosa * with basification of NH_4_OH	[16]
** 18 **	caryopterisines H	* Caryopteris glutinosa * with basification of NH_4_OH	[16]
** 19 **	caryopterisines I	* Caryopteris glutinosa * with basification of NH_4_OH	[16]
** 20 **	Oxerine	* Caryopteris glutinosa * with basification of NH_4_OH	[16]
** 21 **	(5aR*,6S*,10S*,11R*,11aR*)-10,11a-dimethyl-6,7,9,10,11,11a-hexahydro-5H-6,11-epoxycyclopenta [6,7]azuleno[1,2-c]pyridin-5,8(5aH)-dione	* Caryopteris mongolica * Bunge with basification of NH_4_OH	[17]
** 22 **	(5aR*,6S*,7aR*,8S*,11aR*)-10-hydroxy-7a,11a-dimethyl-5a,6,7,7a,8,11a-hexahydro-5H-6,8-epoxycyclopenta[6,7]azuleno[1,2-c]pyridin-5-one	* Caryopteris mongolica * Bunge with basification of NH_4_OH	[17]
** 23 **	(6S*,6aR*,11R*,11aS*)-6a-hydroxy-11,11a-dimethyl-6,6a,11,11a-tetrahydro-5H-6,11-methanopyrido[3′,4′:4,5]cyclopenta[1,2-h]isoquinolin-5-one	* Caryopteris mongolica * Bunge with basification of NH_4_OH	[17]
** 24 **	caryopterisines C	* Caryopteris glutinosa * with basification of NH_4_OH	[16]
** 25 **	caryopterisines D	* Caryopteris glutinosa * with basification of NH_4_OH	[16]
** 26 **	caryopterisines E	* Caryopteris glutinosa * with basification of NH_4_OH	[16]
** 27 **	(5R*,5aR*,10bS*,11R*)-5-hydroxy-10b,11-dimethyl-5,5a,10b,11-tetrahydro-6H-5,11-methanopyrido[3′,4′:3,4]cyclopenta[1,2-g]isoquinolin-6-one (±)-caryopterisines B	* Caryopteris mongolica * Bunge with basification of NH_4_OH *Caryopteris glutinosa* with basification of NH_4_OH	[17,18]
** 28 **	(±)-caryopterisines A	* Caryopteris glutinosa * with basification of NH_4_OH	[18]

(5S*,7R*)-7-Ethoxy-6,7-dihydro-7-methyl-5H-cyclopenta[c]pyridin-5-ol (**15**), (5aR*,6S*,10S*,11R*,11aR*)-10,11a-dimethyl-6,7,9,10,11,11a-hexahydro-5H-6,11-epoxycyclopenta[6,7]azuleno[1,2-c]pyridin-5,8(5aH)-dione (**21**), (5aR*,6S*,7aR*,8S*,11aR*)-10-hydroxy-7a,11a-dimethyl-5a,6,7,7a,8,11a-hexahydro-5H-6,8-epoxycyclopenta[6,7]azuleno[1,2-c]pyridin-5-one (**22**), (6S*,6aR*,11R*,11aS*)-6a-hydroxy-11,11a-dimethyl-6,6a,11,11a-tetrahydro-5H-6,11-methanopyrido[3′,4′:4,5]cyclopenta[1,2-h]isoquinolin-5-one (**23**), and (5R*,5aR*,10bS*,11R*)-5-hydroxy-10b,11-dimethyl-5,5a,10b,11-tetrahydro-6H-5,11-methanopyrido[3′, 4′:3, 4]cyclopenta[1,2-g]isoquinolin-6-one (**27**) were isolated from the aerial parts of the Mongolian medicinal plant *Caryopteris mongolica* Bunge (Lamiaceae) with basification of NH_4_OH [17]. Compounds **15**, **21**–**23**, **27** were all reported with their relative configurations in this paper.

Subsequently, (±)-caryopterisines B (**27**) and A (**28**) with a 6/5/5/5/6 pentacyclic ring system were identified as racemates from *Caryopteris glutinosa* Rehder (Lamiaceae) [18]. Additionally, each of them underwent chiral HPLC examination, and computed ECD or X-ray diffraction analysis was used to determine their absolute configurations. In cell-based estrogen biosynthesis experiments, it was discovered that both **27** and **28** moderately reduced the manufacture of estrogen E2. Additionally, they moderately decreased kynurenine production by inhibiting indoleamine 2,3-dioxygenase [18]. It is possible for compound **28** to only slightly suppress interleukin-1*β* release [18].

(*S*)-7-Methylene-6,7-dihydro-5*H*-cyclopenta[*c*]pyridine-5-ol (**10**), caryopterisines F–I (**16**–**19**), oxerine (**20**), together with caryopterisines C–E (dimeric pyridine-containing alkaloids, **24**–**26**) were characterized from *C. glutinosa*. The unprecedented dimers (**24**–**26**) may be biosynthetically or chemically transformed from a Diels–Alder reaction, a following aromatization rearrangement reaction, or a subsequent Baeyer–Villiger oxidation, and a set of following reactions. Inhibition activities of the nine alkaloids on collagen accumulation through a cell-based assay were carried out. Caryopterisines C (**24**) was indicated to be a potential lead compound with antifibrotic activities [16].

### 2.2. Pyridine Alkaloids Derived from Iridoids (Figure 3, Table 2, Type II)

4-Methylpyridine derivatives are directly ammoniated and aromatized from 4-methyliridoids. Eleven monomers (**29**–**39**) and two dimers (**40**–**41**) from plants were summarized in this class.

**Figure 3:**
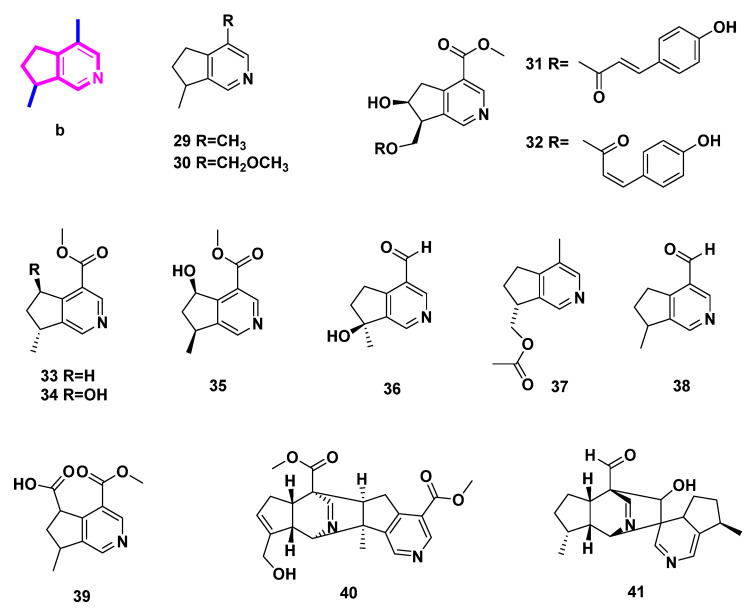
Structures of pyridine alkaloids derived from iridoids (Type II).

The volatile essential oil of *Valeriana officinalis* contains the chemicals actinidine (**29**) and valerianine (**30**), which are also present in a variety of iridoid-rich plants and insect species [19]. Similar to nepetalactone from catnip (*Nepeta cataria*), which is likewise a pheromone for insects and a defense component of rove beetles for several staphylinid species, the alkaloid (**29**), a product of two species of dolichoderine ants in the genus Conomyrma, attracts cats [19]. Actinidine (**29**), a psychotropic alkaloid that disrupts GABA-ergic metabolism and acts as an agonist on benzodiazepine receptors, causes an allosteric modification of the GABA-receptor proteins [19].

Previously, lysine and quinolinic acid were thought to be the biosynthesis precursors of actinidine [19]. Here, we believe that IGs are more likely to be the precursors of actinidine, which are similar to that of monoterpene indole alkaloids [20].

Additionally, two ant species, *Tapinoma melanocephalum* (Formicidae) and I*ridomyrmex anceps* Roger (Formicidae), as well as two plant species, *Actinidia polygama* Maxim (Actinidiaceae) and *Nepeta cataria* L. (Lamiaceae), all underwent heat induction to produce actinidine (**29**) [21].

**Table 2:** Pyridine alkaloids derived from iridoids (Type II).

Number	Compound Name	Source	Reference
** 29 **	actinidine	*Actinidia polygama*; *Valeriana officinalis*	[19,21]
** 30 **	valerianine	* Valeriana officinalis *	[19]
** 31 **	trans- *p*-coumaroyl -9-cantleyine	* Ceolospermum billardieri *	[13]
** 32 **	cis- *p*-coumaroyl -9-cantleyine	* Ceolospermum billardieri *	[13]
** 33 **	deoxyrhexifolin	*Castilleja rbexifolia*; A purported hybrid of *Castilleja rbexifolia* and *Castilleja miniata*	[22]
** 34 **	rhexifoline	A purported hybrid of *Castilleja rbexifolia* and *Castilleja miniate*; Transform of Penstemonoside	[22,23]
** 35 **	cantleyine II	* Strychnos trinervis *	[24]
** 36 **	euphrosine	* Orthocarpus sp. *	[25]
** 37 **	10-acetoxy-actinidine	* Argylia radiata *	[26]
** 38 **	(+)-boscbniakine	* Penstemon whippleanus *	[14]
** 39 **	carbomethoxypedicularine	* Penstemon whippleanus *	[14]
** 40 **	a MTPA Dimer	transformation from geniposide	[27]
** 41 **	lindeniamine	transformation from *Lindenia austro-caledonica* Brongn	[28]

Extracts of *Ceolospermun billardieri* basified by NH_4_OH could afford not only compounds **5**, **8** and **9**, but also *trans* and *cis p*-coumaroyl -9-cantleyine (**31** and **32**) [13]. Similarly, rhexifoline (**33**) and deoxyrhexifoline (**34**) were two alkaloids isolated from the blossoms and seeds of *Castilleja rhexifolia* with basification of NH_4_OH [22]. Additionally, rhexifoline (**33**) was transformed from penstemonoside by means of treatment with NH_3_ [22,23]. Cantleyine II (**35**), a calcium channel inhibitor with properties akin to those of verapamil and nifedipine, was isolated from the root bark of *Strychnos trinervis* (a member of the family Loganiaceae). It was postulated that cantleyine II inhibits Ca^2+^ influx through voltage-gated Ca^2+^ channels, causing a reversible but nonselective spasmolytic activity on the vascular and visceral smooth muscles [24].

Euphrosine (**36**) was isolated from *Ortbocarpus luteus*. Compound **36** could be isolated with basification by NaOH/Na_2_CO_3_ (low yield: 0.003%) or aqueous NH_3_ (higher yield: 0.03%) [25]. Meanwhile, 10-Acetoxy-actinidine (**37**), whose structure was elucidated by Mass spectrometry and ^1^H NMR data, was isolated from the root of *Argylia radiata* (L.) D. Don [26]. Additionally, its structure was biogenetically related to iridoids present in this plant.

(+)-Boscbniakine (**38**) and carbomethoxypedicularine (**39**) were characterized from *Castilleja miniate* together with compounds **11**, **12**, and **13** by means of basification with NH_4_OH [14]. A MTPA dimer (**40**), a Diels–Alder adduct, was transformed from geniposide, which is an iridoid glycoside, reacting with *β*-glucosidase and aqueous NH_4_OAc [27]. Lindeniamine (**41**), another dimer, was isolated from *Lindenia austro-caledonica* Brongn (Rubiaceae), when ammonia was used in extraction [28].

### 2.3. Pyridine Alkaloids Derived from Hemiacetal Secoiridoids (Figure 4, Table 3, Type III)

In this section, five pyridine compounds **42**–**46** derived from the hemiacetal secoiridoid were described with the pyrano–pyridine ring. Compounds **42**–**44** and **46** were isolated from plants, while compound **45** was obtained through microbial transformation of an IG substrate.

**Table 3:** Pyridine alkaloids derived from hemiacetal secoiridoids (Type III).

Number	Compound Name	Source	Reference
** 42 **	(–)-vincapyridine A	* Vinca major *	[29]
** 43 **	(–)-vincapyridine B	* Vinca major *	[29]
** 44 **	(–)-vincapyridine C	* Vinca major *	[29]
** 45 **	gentianal	transformation of gentiopicroside	[30]
** 46 **	jasminin	transformation of secoiridoid glucosides from *Ligustrum vulgare* L.	[31]

**Figure 4:**
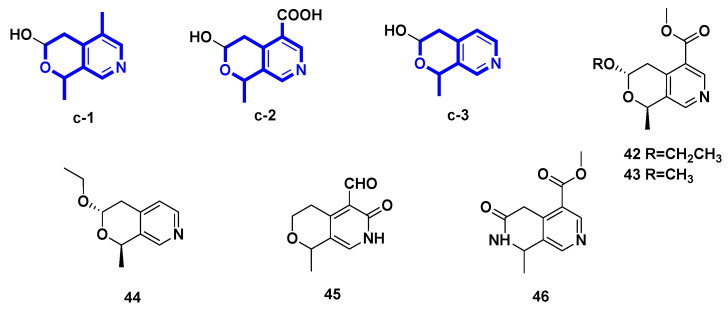
Pyridine alkaloids derived from hemiacetal secoiridoid (Type III).

(−)-Vinmajpyridines A–C (**42**–**44**) were isolated from the aerial parts of *Vinca major* (Apocynaceae) cultivated in Pakistan [29]. Gentianal (**45**) was yielded from the biotransformation of gentiopicroside by the asexual mycelia of *Cordyceps sinensis* [30]. However, jasminin (**46**) was isolated by treating secoiridoid glucosides from fruits of *Ligustrum vulgare* L. with H_2_SO_4_ and subsequently NH_3_.

### 2.4. Pyridine Alkaloids Derived from Secoiridoids (Figure 5, Table 4, Type IV)

In this class, pyridines were directly derived from secoiridoid through ammonization and aromatization as chemical transformations. There were five monomers and four dimers.

Methyl 5-ethylnicotinate (**47**), methyl 5-ethyl-4-methyl-nicotinate (**48**), *p*-hydroxy-*β*-phenethyI5-ethyl-3-methoxycarbonyl-4-pyridinylacetate (**49**), and 3,4-dihydroxy-*β*-phenylethyl-5-ethyl-3-methoxycarbonyl-4-pyridinylacetate (**50**) are pyridine alkaloids transformed from secoiridoid glucosides in *Ligustrum vulgare* L. [31,32,33], in the same way as **36**. Meanwhile, *p*-hydroxy-*β*-phenetyl-PD-glucopyranoside 6-(5-ethyl-3-methoxycarbonyl)-4-pyridineacetate (**51**) [32], 4-methyl-5,5’-[(1-methyltrimethylen)di](methylnicotinate) (**52**), 4.4’-bis-methyl-5,5’-[(l-methyltrimethylen)di](methylnicotinate) (**53**), methyl 3,3′-bis-methoxycarbony1-5,5′-[(1-methyltrimethylene)di]-4,4′-bis-piridinylacetate (**54**), and methyl 5-[3-(3-methoxycarbonyI-5-pyridinyl)-l-methylpropyl]-3-methoxycarbonyl-4-pyridinylacetate (**55**) [33,34] were yielded in the same way.

**Figure 5:**
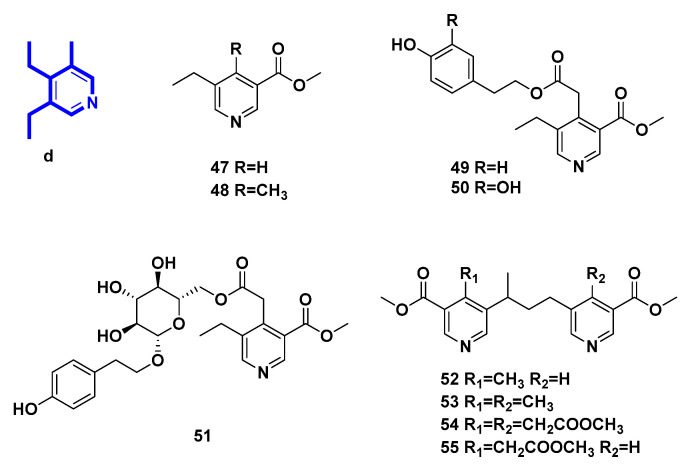
Structures of pyridine alkaloids derived from secoiridoids (Type IV).

**Table 4:** Pyridine alkaloids derived from secoiridoids (Type IV).

Number	Compound Name	Source	Reference
** 47 **	methyl 5-ethylnicotinate	transformation of secoiridoid glucosides from *Ligustrum vulgare* L.	[31]
** 48 **	methyI5-ethyl-4-methyl-nicotinate	transformation of secoiridoid glucosides from *Ligustrum vulgare* L.	[31,32,33]
** 49 **	p-hydroxy-*β*-phenethyI5-ethyl-3-methoxycarbonyl-4-pyridinylacetate	transformation of secoiridoid glucosides from *Ligustrum vulgare* L.	[31,32,33]
** 50 **	3,4-dihydroxy-*β*-phenylethyl-5-ethyl-3-methoxycarbonyl-4-pyridinylacetate	transformation of secoiridoid glucosides	[33]
** 51 **	p-hydroxy-*β*-phenetyl-PD-glucopyranoside 6-(5-ethyl-3-methoxycarbonyl)-4-pyridineacetate	transformation of secoiridoid glucosides from *Ligustrum vulgare* L.	[32]
** 52 **	4-methyl-5,5′-[(1-methyltrimethylen)di](methylnicotinate)	transformation of secoiridoid glucosides from *Ligustrum vulgare* L.	[33,34]
** 53 **	4.4′-bis-methyl-5,5′-[(l-methyltrimethylen)di](methylnicotinate).	transformation of secoiridoid glucosides from *Ligustrum vulgare* L.	[33,34]
** 54 **	methyl 3,3′-bis-methoxycarbony1-5,5′-[(1-methyltrimethylene)di]-4,4′-bis-piridinylacetate	transformation of secoiridoid glucosides	[33]
** 55 **	methyl 5-[3-(3-methoxycarbonyI-5-pyridinyl)-l-methylpropyl]-3-methoxycarbonyl-4-pyridinylacetate	transformation of secoiridoid glucosides	[33]

### 2.5. Pyridines Alkaloids Derived from Lactone Secoiridoids (Figure 6, Table 5, Type V)

Here, three compounds are summarized. All of them possessed the pyridine fused *δ*-valerolactone skeleton and could be obtained from chemical transformation of IGs. Meanwhile, compound **57** could be yielded from biotransformation by fungi.

Gentianine (**56**) and (*Z*)-5-ethylidene-8-hydroxy-3,4,5,6,7,8-hexahydropyrano[3,4-c]pyridine-1-one (**57**) could be yielded in the same way as gentianal (**45**) [30,35]. Moreover, compound **57** could also be biotransformed from swertiamarin by *Aspergillus niger* [35]. Oliveramine (**58**) could be yielded in the same way as **52**–**55** [34]. Gentianine (**56**) was also isolated from *Gentiana kirilowii,* and many other *Gentiana* and *Swertia* species of the Gentianaceae family. Gentianine, which works as a CNS stimulant in low doses but transforms into a paralytic in greater levels, had its antipsychotic profile examined [36].

**Figure 6:**
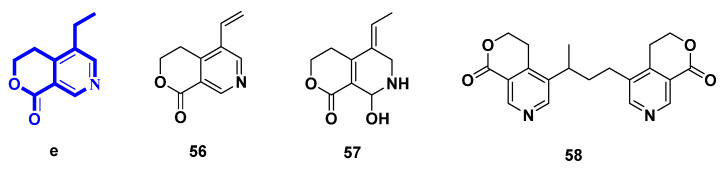
Structures of pyridines alkaloids derived from lactone secoiridoids (Type V).

**Table 5:** Pyridines alkaloids derived from lactone secoiridoids (Type V).

Number	Compound Name	Source	Reference
** 56 **	gentianine	transformation of gentiopicroside	[30]
** 57 **	(*Z*)-5-ethylidene-8-hydroxy-3,4,5,6,7,8-hexahydropyrano[3,4-c]pyridine-1-one	biotransformation of gentiopicroside by asexual mycelia of *Cordyceps sinensis*; biotransformation of swertiamarin by *Aspergillus niger*	[30,35]
** 58 **	oliveramine	transformation from *Gentiana olivieui*	[34]

### 2.6. Phenyl-Substituted Cyclopenta[c]pyridine Derivatives (Figure 7, Table 6, Type VI)

To date, twenty-six phenyl-substituted cyclopenta[c]pyridine compounds have been found exclusively in the genus Ganoderma. It was deduced that these uncommon alkaloids are probably biosynthesized via meroterpenoid and threonine [37]. In this case, there might be exclusive enzymes in this genus. Phenyl-substituted cyclopenta[c]pyridine may be an important chemtaxonomic characteristic to distinguish Ganoderma from other fungi.

Ganocochlearines A−I (**69**, **70**, **59**, **61**–**66**), ganoapplanatumine B (**60**), sinensine E (**67**), and lucidimine C (**68**), most of which possessed a phenyl-substituted cyclopenta[c]pyridine skeleton, were obtained from the fruiting bodies of the fungus *Ganoderma cochlear* [37,38]. Among them, compounds **59**–**68** were all alkaloid enantiomers. Two alkaloid enantiomers [(±)-**71** and (±)-**67**] were identified from *Ganoderma luteomarginatum*, a rare species in the genus Ganoderma, possessing the phenyl-substituted cyclopenta[c]pyridine skeleton [39]. To the best of our knowledge, only the genus Ganoderma has been associated with this type of skeleton in the natural world. Racemic cyclopenta[c]pyridines may indicate the presence of unique enzymes in Ganoderma.

Five alkaloids, sinensine A-E (**72**–**76**), were deduced from the fruiting bodies of *Ganoderma sinense* Zhao, Xu *et* Zhang, which is a traditional Chinese medicine [40,41]. With a protection rate of 70.90% and an EC_50_ value of 6.23 µmol/L, Sinensine A (**72**) exhibits activity in preventing the damage caused by hydrogen peroxide oxidation on human umbilical cord endothelial cells (HUVEC) [41].

The fruiting bodies of *Ganoderma applanatum* included the racemic ganoapplanatumines A (**77**), B (**78**) and *epi*-ganoapplanatumine B (**79**) [42].

The *Ganoderma lucidum* fruiting bodies contained the four polycylic alkaloids lucidimine A–D (**80**–**83**) [43]. Additionally, *Ganoderma lucidum* produced ganocochlearine A (**69**), which was separated and characterized. This compound demonstrated impressive neuroprotection with an EC_50_ value of 2.49 ± 0.12 μM and effective anti-inflammation with an IC_50_ value of 4.68 ± 0.09 μM [44]. *Ganoderma australe*’s fruiting bodies also yielded ganoochlearine A (**69**), which was able to considerably protect SH-SY5Y cells from glutamate-induced neural excitotoxicity at 10 μM and nearly reach its maximum efficacy at 20 μM [45].

The *Ganoderma calidophilum* fruiting body’s EtOAc extract was used to isolate compounds **84** (ganocalicine A) and **81** (lucidimine B/ganocalicine B). Compounds **81** and **84** strongly decreased the synthesis of IL-4 and LTB_4_ by RBL-2H3 cells in response to antigen stimulation and inhibited the activity of *β*-hexosaminidase (IC_50_ 9.14 and 9.44 µM, respectively). This suggests that **81** and **84** have anti-allergic properties [46].

**Table 6:** Phenyl-substituted Cyclopenta[c]pyridine Derivatives (Type VI).

Number	Compound Name	Source	Reference
59	ganocochlearine C	*Ganoderma cochlear*	[37,38]
60	ganoapplanatumine B	*Ganoderma cochlear*	[37,38]
61	ganocochlearine D	*Ganoderma cochlear*	[37,38]
62	ganocochlearine E	*Ganoderma cochlear*	[37,38]
63	ganocochlearine F	*Ganoderma cochlear*	[37,38]
64	ganocochlearine G	*Ganoderma cochlear*	[37,38]
65	ganocochlearine H	*Ganoderma cochlear*	[37,38]
66	ganocochlearine I	*Ganoderma cochlear*	[37,38]
67	sinensine E	*Ganoderma cochlear*	[37,38]
68	lucidimine C	*Ganoderma cochlear*	[37,38]
69	ganocochlearine A	*Ganoderma cochlear, Ganoderma lucidum, Ganoderma austral*	[37,38,44,45]
70	ganocochlearine B	*Ganoderma cochlear*	[37,38]
71	6-hydroxyganocochlearine A	*Ganoderma luteomarginatum*	[39]
72	sinensine A	*Ganoderma sinense*	[40]
73	sinensine B	*Ganoderma sinense*	[40]
74	sinensine C	*Ganoderma sinense*	[40]
75	sinensine D	*Ganoderma sinense*	[40]
76	sinensine E	*Ganoderma sinense*	[40]
77	ganoapplanatumine A	*Ganoderma applanatum*	[42]
78	ganoapplanatumine B	*Ganoderma applanatum*	[42]
79	epi-ganoapplanatumine B	*Ganoderma applanatum*	[42]
80	lucidimine A	*Ganoderma lucidum*	[43]
81	lucidimine B/ganocalicine B	*Ganoderma lucidum, Ganoderma calidophilum*	[43,46]
82	lucidimine C	*Ganoderma lucidum*	[43]
83	lucidimine D	*Ganoderma lucidum*	[43]
84	ganocalicine A	*Ganoderma calidophilum*	[46]

**Figure 7:**
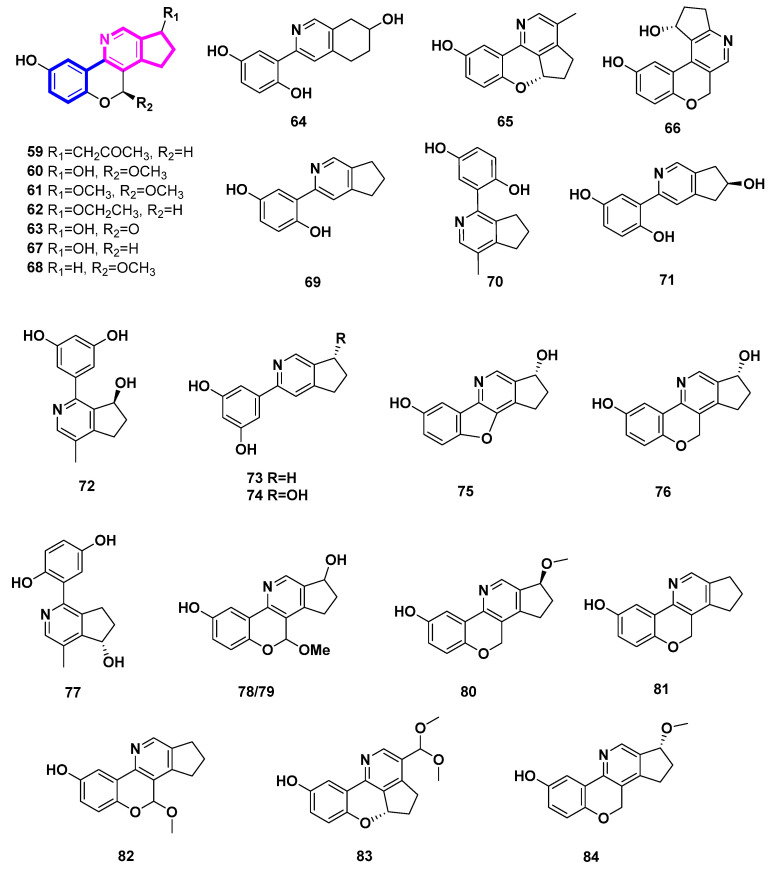
Structures of phenyl-substituted cyclopenta[c]pyridine derivatives (Type VI).

## 3. Generation of MTPAs and Their Activities

Based on the available reports of MTPAs and cyclopenta[c]pyridines, these alkaloids have the five following origins: Firstly, these natural products are obtained without using nitrogen containing chemicals (concentrated ammonia, ammonium salts, etc.) in the extraction and separation process [10,19,26,29,32,34,39,47,48,49,50,51,52,53]; Secondly, some are products of the microbial biotransformation of iridoid glycosides [30,35,54,55,56,57]. Thirdly, these products may be artificial or natural, using nitrogen-containing chemicals in the extraction and separation process [11,12,13,14,22,28,58]. Fourthly, these products can be directly derived from the chemical transformation of iridoid glycosides with nitrogen-containing chemicals [23,25,27,31,33]. Fifthly, phenyl-substituted cyclopenta[c]pyridines only existed in the genus Ganoderma due to the special enzymes in these mushrooms [37,38,39,40,41,42,43,44,46].

In this paper, we summarized the chemical transformation, biotransformation, chemical synthesis, and bioactivities of the MTPAs and cyclopenta[c]pyridines. This could help us obtain a clear view of the chemistry and biology of MTPAs and cyclopenta[c]pyridines.

### 3.1. MTPAs Yielded by Chemical Transformation of IGs

**Scheme 3:**
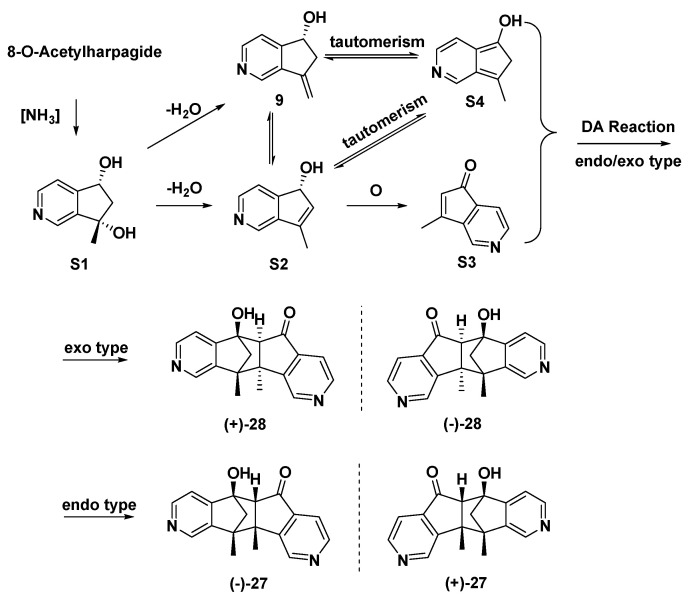
Proposed genetic pathway for (±)-**27** and (±)-**28**.

Both (±)-**27** and (±)-**28** were supposed to be derived from 8-*O*-acetylharpagide, which is found in great quantities in the plant *C. glutinosa*. Naturally or in the presence of ammonia, 8-*O*-acetylharpagide may be converted to MTPAs (Scheme 3, intermediates: **S1**–**S3**, **9**, and **S4**). The key step in their formation might be the Diels–Alder reaction of intermediates **S3** and **S4** to afford (**±**)-**28** (*exo* type) and (**±**)-**27** (*endo* type).

**Scheme 4:**
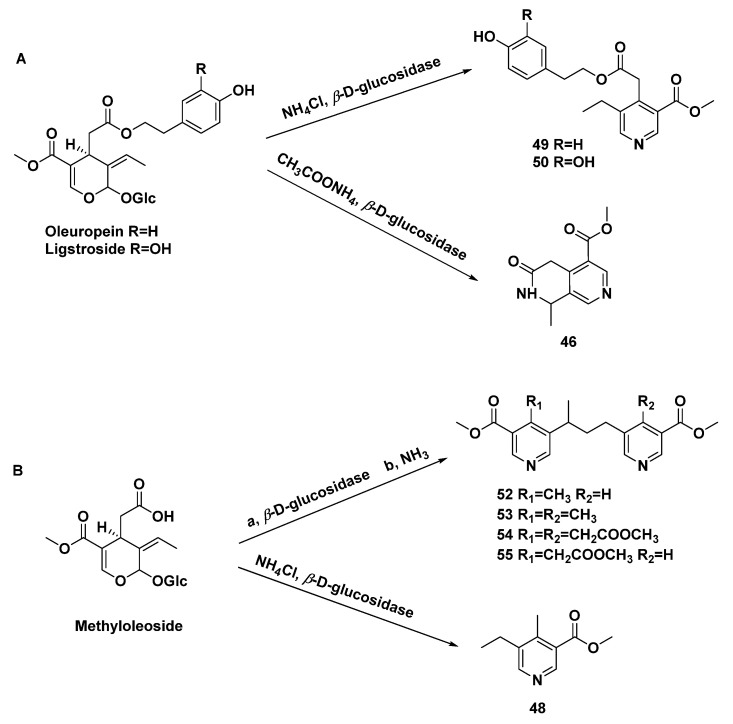
Amination of secoiridoid glycosides ((**A**), oleuropein and ligstroside as substrates; (**B**), methyloleoside as substrates) to give monomeric pyridine, dimeric pyridine, and naphthyridine alkaloids.

The selective amination of secoiridoid glycosides afforded monomeric pyridine alkaloids (**48**–**50**), dimeric pyridine alkaloids (**52**–**55**), and naphthyridine alkaloids (**46**), which was catalyzed by *β*-D-glucosidase. [33] (Scheme 4) The secoiridoid glucosides isolated from *Ligustrum vulgare* L. were subjected to 5 % H_2_SO_4_ and NH_3_ (gas) subsequently, affording compounds **46**, **48,** and **49** [31].

**Scheme 5:**
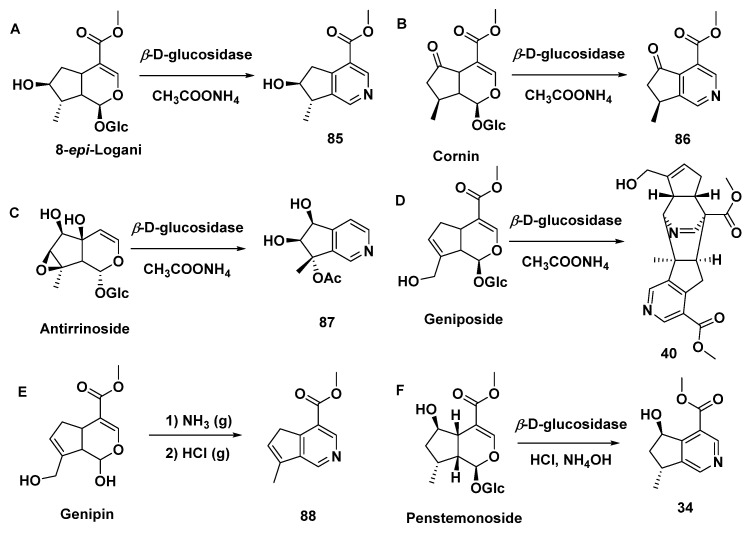
MTPAs produced from IGs.

**Scheme 6:**
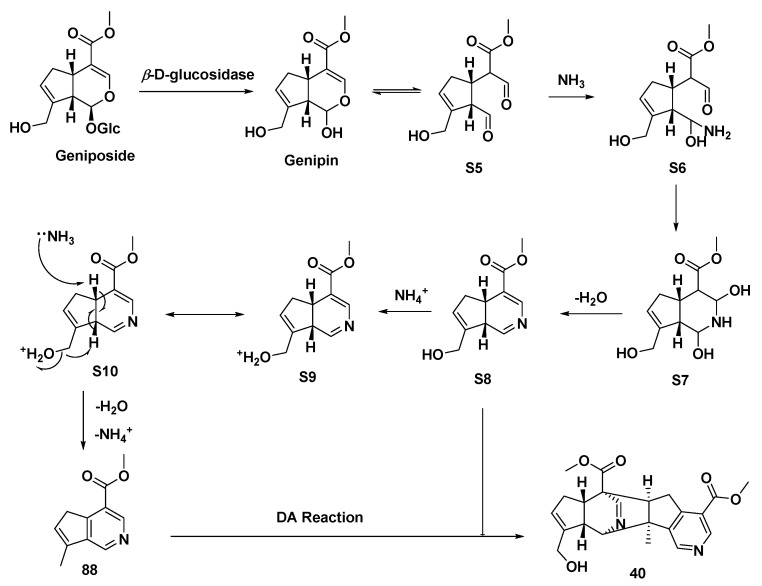
Possible transformation mode to afford a monomeric MTPA (**88**) and the dimeric MTPA (**40**).

A series of iridoid glycosides treated by *β*-D-glucosidase and CH_3_COONH_4_ yielded MTPA monomers [8-*epi*-cantleyine **85** (Type II), corninine **86** (Type II), [7-(*S*)-acetoxy-5,6-(*R*, *S*)-dihydroxy-7(*R*)-methylcyclopenteno[c]pyridine **87** (Type I), racemigerine **88** (Type II)] and a dimer (**40**), accompanied by ammonium acetate or NH_3_ (g)/HCl (g) (Scheme 5A–E) [27]. The biogenic synthetic pathways of compound **40** are speculated upon in Scheme 6 [27], while the electron transfer of intermediate **S10** is lightly revised, differing from the original article.

Penstemonoside was chemically converted to rhexifoline (**34**), which is shown in Scheme 5F. [23] At the same time, compound **34** was isolated from a hybrid species of *Castilleja rbexifolia* and *Castilleja miniate* by the same author, although aqueous ammonia was used in the extraction process [22].

**Scheme 7:**
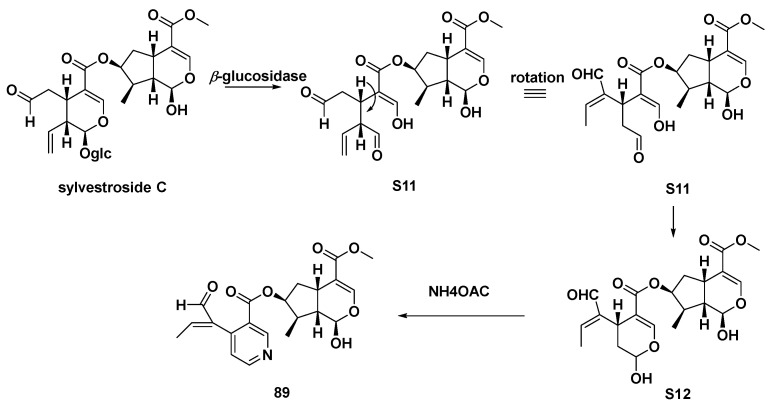
Possible biosynthetic route of compound **89**.

It was found that pterocenoids A (Scheme 7, **89**) and two other iridoid dimers from *Pterocephalus hookeri* exhibited moderate inhibitory activity in the NF-*κ*B pathway. This was also consistent with the application of their botanical source, which is used to treat inflammatory disease in Tibetan herbal medicine. Therefore, it was supposed that such iridoid dimers were effective anti-inflammatory components [48].

### 3.2. MTPAs Generated from the Biotransformation of IGs

It has been reported that iridoid glycosides could be converted into MTPAs by fungi or human intestinal bacteria. Therefore, some researchers argued that the activities and potency of iridoid glycosides are attributed to their conversion to MPTAs [55].

**Scheme 8:**
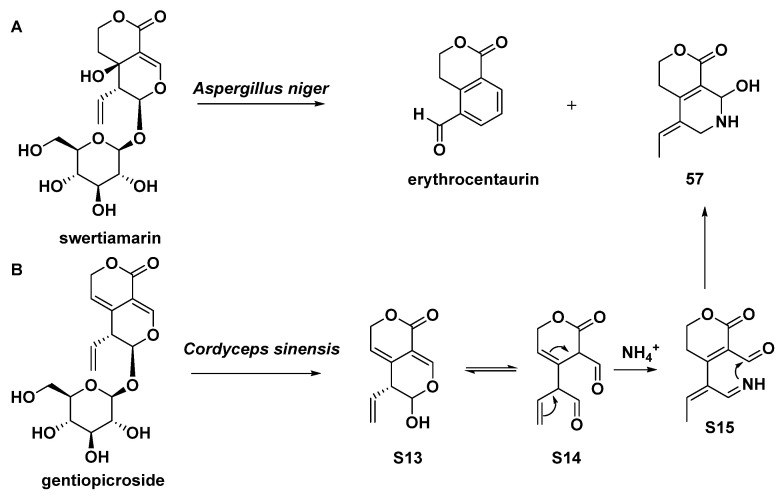
Compound **57** yielded from *Aspergillus niger* (**A**) or *Cordyceps sinensis* (**B**) by means of biotransformation.

As mentioned previously, *Aspergillus niger* could convert swertiamarin to naphthyridine (**57,** Scheme 8). In another case, the asexual mycelium of the fungus *Cordyceps sinensis* was able to convert gentiopicroside into compound **57** as well (Scheme 8) [19].

**Scheme 9:**
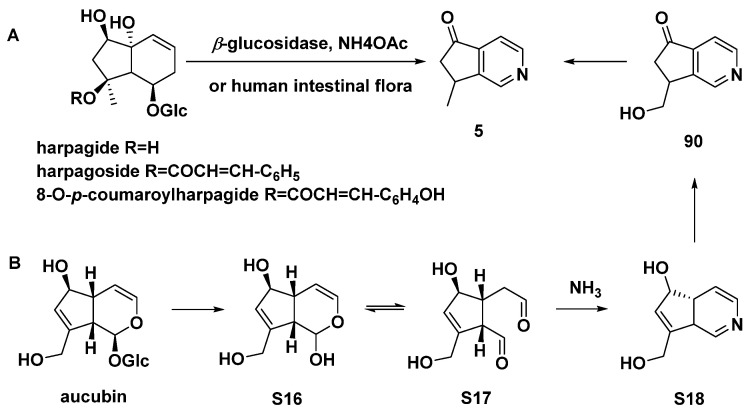
Conversion of three harpagide congeners to aucubinine B (**5**) by *β*-glucosidase in the presence of NH_4_OAc or by human intestinal flora (**A**); possible metabolic processes of aucubin by human intestinal bacteria (**B**).

Harpagide, harpagoside, or 8-O-*p*-coumaroylharpagide could be transformed to aucubinine B (**5**) by human intestinal bacteria (Scheme 9A) [54]. It was reported that aucubin can be converted to aucubinines A (**90,** Type I) and B (**5**) by human intestinal bacteria [57], as shown in Scheme 9B.

In summary, both the chemical transformations and biotransformation of MTPAs form cyclopenta[c]pyridines molecular skeleton after amination of iridoids. Therefore, it is reasonable to speculate that MTPAs could be biosynthesized in a similar way by living organisms.

### 3.3. Chemical Synthesis of MPTAs/Cyclopenta[c]pyridines

In recent years, there have been many preeminent works on the total synthesis of MPTAs, and most of them employed one-step reaction to synthesize the target compounds. The early synthesis products were racemic, which was not reported in this paper. Additionally, all of the recent reports were chiral synthesis works, and are described in chronological order as follows.

**Scheme 10:**
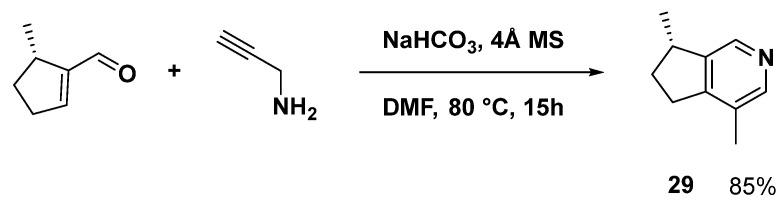
One-step synthesis of the monoterpene natural product, (−)-actinidine (**29**).

In 2019, a straightforward tandem method for synthesizing pyridine derivatives in the absence of metals was described, and the chiral synthesis of (−)-actinidine (**29**) was finished in a single step (Scheme 10) [59].

**Scheme 11:**
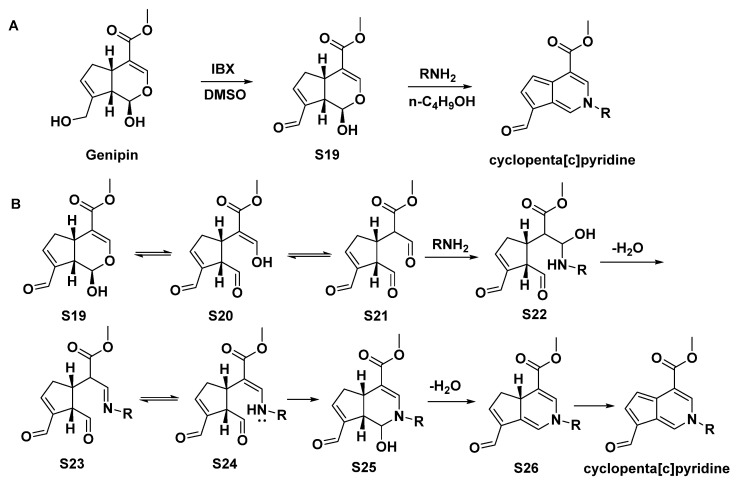
(**A**)**,** Synthesis of cyclopenta[c]pyridine from genipin; (**B**)**,** possible reaction mechanism.

An efficient and economical two-step synthesis of the cyclopenta[c]pyridines was reported, using an iridoid (genipin) as the substrate [2], as shown in Scheme 11. Additionally, the possible reaction mechanism was proposed, such as an aldol reaction. Compounds **91**–**112** were afforded in this way.

**Figure 8:**
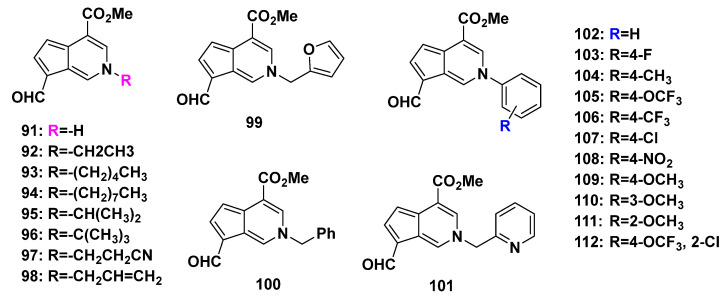
Cyclopenta[c]pyridine products (**91**–**112**) obtained from a concise biomimic access.

Li, L. et al. tested the anti-TMV activity of a series of synthesized cyclopenta[c]pyridine compounds (**91**–**112**, Scheme 11 and Figure 8). All the compounds were non-toxic against *Nicotiana tabacum* L. In subsequent anti-TMV activity tests, most of **91**–**112** showed good activities. Compound **109** [2-(4-methoxyphenyl)] had optimal anti-TMV activity with an inactivation effect of 40.2 ± 4.5%, a curative effect of 44.9 ± 4.0%, and a protection effect of 39.6 ± 2.3% at 500 μg/mL [2].

The insecticidal activities of the synthesized cyclopenta[c]pyridines **91**–**112** were also evaluated. Among those compounds, only compound **112** [2-(2-chloro-4-(trifluoromethoxy) phenyl)] exhibited activity against *Plutella xylostella* comparable to that of cerbinal, which was the natural precursor with an oxygen atom at the 2-position without modification. In view of this, the modification of the cyclopenta[c]pyridine skeleton at the 2-position significantly increased the anti-TMV activity but was not beneficial for insecticidal activity [2].

**Scheme 12:**
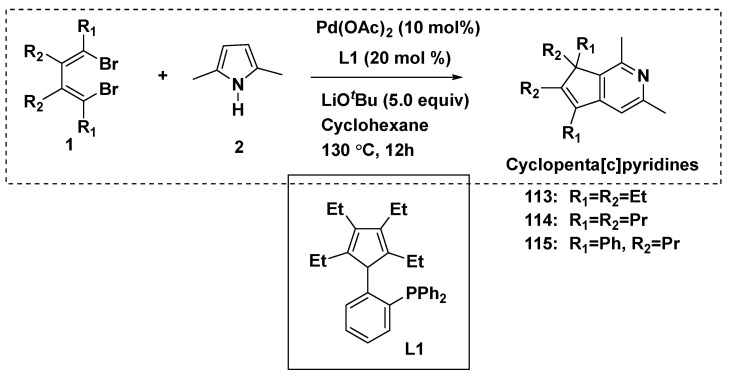
Reactions afforded cyclopenta[c]pyridine derivatives.

The molecular skeleton of cyclopenta[c]pyridines was constructed in a one-step reaction between 1,4-dibromo-1,3-butadienes and 2,5-disubstituted pyrroles, using one-carbon expansion of the pyrrole skeleton to pyridine (Scheme 12) [60]. Additionally, the reaction used Pd(OAc)2 (10%) and cyclopentadiene-phosphine (L1) as the catalysts. Cyclopenta[c]pyridine compounds **113**–**115** were afforded.

**Scheme 13:**
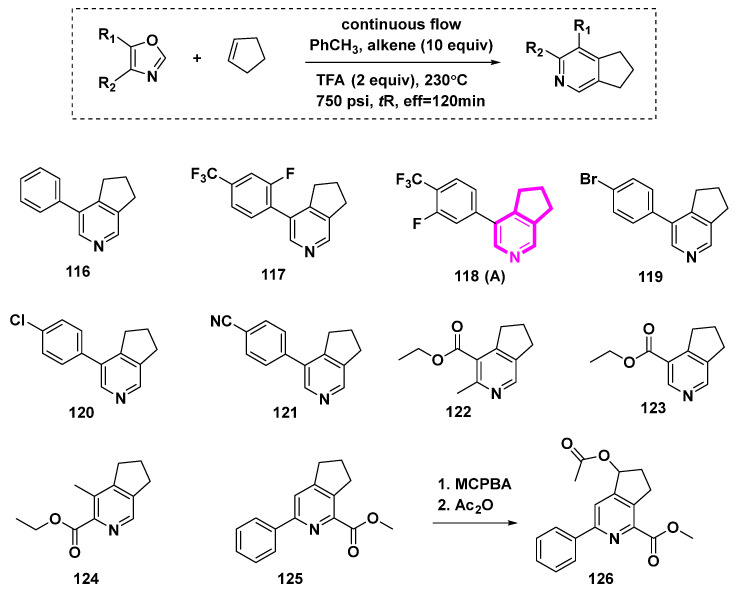
The Kondrat’eva Reaction in Flow: synthesis of annulated pyridines.

The Kondrat’eva reaction was used to prepare annulated pyridines directly in a flow device [4,5]. However, the reaction conditions of high temperature and pressure were needed, as depicted in Scheme 13. Cyclopenta[c]pyridine compounds **116**–**126** were yielded.

**Scheme 14:**
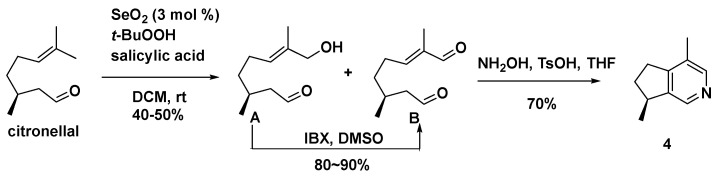
Diastereoselective synthesis of actinidine (**4**).

A divergent retrosynthetic analysis strategy (Scheme 14) was used to synthesize a series of iridoid derivatives, including actinidine (**4**) [61].

**Scheme 15:**
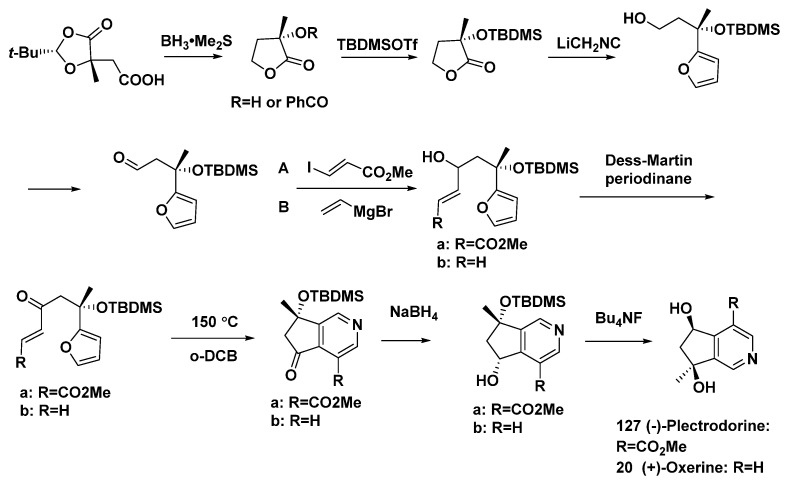
Total synthesis of the monoterpene alkaloids (−)-plectrodorine (**127**) and (+)-oxerine (**20**).

The total synthesis of (−)-plectrodorine (**127**) and (+)-oxerine (**20**) was completed in nine steps [62], using the oxazole–olefin Diels–Alder reaction (Scheme 15).

To sum up, the total synthesis of the molecular skeleton of MTPAs and cyclopenta[c]pyridines is becoming increasingly concise, efficient, economical, and green.

## 4. Conclusions

In total, we categorized six types of MTPAs and cyclopenta[c]pyridines by their origins and structures. MTPAs **1**–**58** and **75**–**90** originated from iridoids as natural, chemically transformed, enzyme catalyzed, or microbial transformed products. Phenyl-substituted cyclopenta[c]pyridine derivatives **59**–**84** were characteristic constituents in the genus Ganoderma and were proposed to be biosynthesized via meroterpenoid and threonine. To date, cyclopenta[c]pyridine compounds **4**, **20**, **29,** and **91**–**127** have been concisely and chirally synthesized. The synthetic methods were concise, green, and productive. Additionally, MPTAs or cyclopenta[c]pyridines have shown potential with antibacterial, insecticidal, antiviral, and anti-inflammatory activities, as reported. It has been suggested that some of the iridoids were activated because of their transformations into MTPAs after ingestion. This was supported by the conversion of iridoids to MTPAs by intestinal microorganisms [55]. Meanwhile, iridoids are widely distributed and abundant in plants.

We believe that this paper will contribute to the further investigation of MTPAs and cyclopenta[c]pyridines regarding their origin, synthesis, and bioactivities. Therefore, further investigations of the chemistry and biology of MTPAs and cyclopenta[c]pyridines are expected.

## Data Availability

All data included in this review are available upon request by contact with the corresponding author.
